# ISMB/ECCB 2025 Proceedings

**DOI:** 10.1093/bioinformatics/btaf271

**Published:** 2025-07-15

**Authors:** Karsten Borgwardt, Tijana Milenković

**Affiliations:** Department of Machine Learning and Systems Biology, Max Planck Institute of Biochemistry, 82152 Martinsried, Germany; Department of Computer Science and Engineering, University of Notre Dame, Notre Dame, IN 46556, United States

## Abstract

This editorial describes the review and selection process of full-length, original research papers submitted to the Proceedings track of the ISMB/ECCB 2025 conference.

## The paper review and selection process

This special issue of the *Bioinformatics* journal serves as the Proceedings of the joint 33rd annual conference on Intelligent Systems for Molecular Biology and 24th annual European Conference on Computational Biology (ISMB/ECCB 2025). This joint meeting took place during 20–24 July 2025, in Liverpool, UK. ISMB/ECCB is the leading international forum for presenting new research results, disseminating methods, and facilitating scientific discussions among researchers in the field of computational biology. The ISMB portion of the joint meeting is the flagship conference of the International Society for Computational Biology (ISCB), and the largest event of the computational biology community. The 2025 meeting was run as a hybrid conference, with the online participants from around the world complementing the on-site participants.

A total of 395 Proceedings submissions were made to ISMB/ECCB 2025. During the submission process, the authors could indicate up to three preferred Communities of Special Interest (COSIs; Table 1; https://www.iscb.org/cosis/info) that would provide the best forum for presentation of their paper if selected to the conference Proceedings. All 371 papers adhering to the submission instructions were thoroughly reviewed, receiving 4.65 reviews per paper on average, for a total of 1727 returned reviewer reports. For the review purpose, the submitted manuscripts were assigned to one of 11 scientific areas (Table 2) according to authors’ preferences based on their papers’ research topics, allowing for minor adjustments, e.g. to avoid conflicts of interest.

**Table 1.** COSI distribution of the accepted Proceedings papers.

COSI	Number of papers
3DSIG	4
BioInfo-Core	0
BioVis	0
BOKR	1
BOSC	0
CAMDA	1
CompMS	0
CSI	1
Education	2
EvolCompGen	4
Function	2
HitSeq	9
iRNA	1
JPI	0
Microbiome	4
MLCSB	10
NetBio	3
RegSys	9
SysMod	1
Text Mining	1
TransMed	3
VarI	0
General Comp Bio	11

**Table 2.** Thematic areas of Proceedings submissions.^a^

Area	Area Chairs	#Sub.	#Acc.	Acc.%
Bioinformatics Education and Citizen Science	Russell Schwartz, Carnegie Mellon University, USA Jérôme Waldispühl, McGill University, Canada	2	2	100%
Bioinformatics of Microbes and Microbiomes	Nicola Mulder, University of Cape Town, South Africa Mihai Pop, University of Maryland, USA	22	3	14%
Biomedical Informatics	Niko Beerenwinkel, ETH Zurich, Switzerland Giulio Caravagna, University of Trieste, Italy Jenna Wiens, University of Michigan, USA	79	11	14%
Equity and Diversity in Computational Biology Research	Larry Hunter, University of Chicago, USA Alejandra Medina Rivera, Universidad Nacional Autónoma de México, Mexico	3	1	33%
Evolutionary, Comparative, and Population Genomics	Flora Jay, Université Paris-Saclay, France Erin Molloy, University of Maryland, USA	17	3	18%
Genome Sequence Analysis	Laurent Jacob, CNRS, Sorbonne Université, France Tobias Marschall, Heinrich Heine University Düsseldorf, Germany	51	11	22%
Macromolecular Sequence, Structure, and Function	Jianlin Cheng, University of Missouri, USA Mark Wass, University of Kent, UK	72	15	21%
Privacy and Security for Computational Biology	Michael Baudis, University of Zurich, Switzerland Kana Shimizu, Waseda University, Japan	4	1	25%
Regulatory and Functional Genomics	Kimberly Glass, Harvard Medical School, USA Saurabh Sinha, Georgia Institute of Technology, USA	62	10	16%
Systems Biology and Networks	Anaïs Baudot, Aix Marseille Université, France Nataša Pržulj, Mohamed bin Zayed University of Artificial Intelligence, United Arab Emirates	43	5	12%
General Computational Biology	Gary Bader, University of Toronto, Canada Alberto Paccanaro, Fundação Getúlio Vargas, Brazil & Royal Holloway, University of London, UK	40	5	13%

a For each area, the table lists the Area Chairs, the number of submissions, the number of accepted papers, and the acceptance rate. Note that the *General Computational Biology* area was intended for submissions on emerging topics or for those manuscripts that did not fit well in the other areas.

The review process was overseen by the Proceedings Chairs (this editorial’s authors). Each area had Area Chairs (ACs) assigned to it (Table 2), who handled the review process within their respective area. The ACs were nominated by the ISMB/ECCB 2025 Steering Committee or by COSIs. The ACs were responsible for recruiting the Program Committee for their area. Reviewers (Program Committee members, who potentially appointed subreviewers to their assigned papers) judged the papers based on the novelty of computational approaches, relevance of biological questions, importance of biological insights, clarity of presentations, expected impact, compatibility with the conference format, and, importantly, correctness, completeness, and reproducibility of the studies. After submitting their reviews, the reviewers had the opportunity to discuss the papers and refine the reviews. The ACs facilitated these discussions. Conditional/provisional acceptance decisions were made based on the reviews, the outcome of these discussions, and the outcome of another, detailed discussion between the ACs and Proceedings Chairs.

Of all Proceedings submissions, 69 were provisionally accepted, conditioned on the authors properly addressing the ACs’ and reviewers’ comments during a 2-week revision period. In a few cases, the authors had to be reminded to release their source code alongside their manuscript or clarify some aspects of their evaluations. This year, all but two of the revised conditionally accepted papers were subsequently judged to have adequately addressed the ACs’ and reviewers’ concerns. These 69−2=67 papers were thus officially accepted in the end for presentation at ISMB/ECCB 2025 and publication in the conference Proceedings, resulting in an overall acceptance rate of 67/395=17%. The acceptance rates for the 11 individual areas are shown in Table 2. The accepted papers were then assigned to COSIs based on the preferences of both authors and COSI organizers (Table 1). The selected papers covered a broad spectrum of topics, spanning 17 of the 23 COSIs (Table 1) and all 11 areas (Table 2). Note that one of the 67 selected papers was voluntarily withdrawn by its authors after the acceptance but before presentation at the conference and publication in the Proceedings.

Throughout the review process, we followed a stringent policy to avoid conflicts of interest. Submissions co-authored by an AC were reassigned to a different area. Submissions with any other type of association to an AC were handled by a different AC. Care was taken not to assign papers to reviewers with an actual or perceived conflict either. The definition of a conflict was as broad as possible, including collaborators (present and past few years), same institution (present, past few years, planned future moves), family relations, advisees/advisors, and any personal conflicts that could cause the appearance of conflict of interest, or that could genuinely interfere with objective reviewing. Finally, as Proceedings Chairs, we refrained from submitting papers to the conference.

We are extremely grateful to the 23 ACs, 406 Program Committee members, and 455 subreviewers for their outstanding efforts in conducting thorough and timely reviews. Their contribution is at the core of the scientific quality of the conference. We also thank ISCB’s Belinda Hanson, Seth Munholland, and Diane Kovats for their support and guidance with handling many logistical aspects, and the ISMB/ECCB 2025 Steering Committee for their expert advise and oversight. We also thank the team at Oxford University Press for producing this special issue.

Moreover, we thank all the authors for submitting their work. These Proceedings would not be possible without the scientific ingenuity of the contributors of all the papers. Despite the efforts taken to ensure fairness and high quality of the paper review and selection process, we acknowledge that the process is necessarily imperfect. Especially given the highly competitive nature of paper selection at ISMB/ECCB 2025, even some very good submissions could not be accepted to the conference. Nonetheless, we hope that all authors received helpful feedback on their work. Finally, we want to thank all the keynote speakers, presenters, and all conference participants.

Thank you all for making this meeting possible, and thus for empowering the ISMB/ECCB community to continue to thrive.

